# Critical illness-induced bone loss is related to deficient autophagy and histone hypomethylation

**DOI:** 10.1186/s40635-015-0052-3

**Published:** 2015-06-21

**Authors:** Helen C Owen, Ineke Vanhees, Jan Gunst, Sophie Van Cromphaut, Greet Van den Berghe

**Affiliations:** Research Group of Intensive Care Medicine, Department of Cellular and Molecular Medicine, KU Leuven, Leuven, Belgium

**Keywords:** Critical Care, Cellular Autophagy, Epigenetics, Osteoclastic Bone Loss

## Abstract

**Background:**

Survivors of critical illness are at increased risk of fractures. This may be due to increased osteoclast formation during critical illness, leading to trabecular bone loss. Such bone loss has also been observed in Paget’s disease, and has been related to deficient autophagy. Deficient autophagy has also been documented in vital organs and skeletal muscle of critically ill patients. The objective of this study was to investigate whether deficient autophagy can be linked to critical illness-induced bone loss.

**Methods:**

Osteoclasts grown *in vitro* and their precursor cells isolated from peripheral blood of critically ill patients and from matched healthy volunteers were analysed for the expression of autophagy genes (SQSTM1, Atg3 and Atg7), and proteins (p62, Atg–5, and microtubule-associated protein light chain 3–II (LC3–II)) and for autophagy and epigenetic signalling factors via PCR arrays and were treated with the autophagy inducer rapamycin. The effect of rapamycin was also investigated at the tissue level in an *in vivo* rabbit model of critical illness.

**Results:**

Many more osteoclasts formed *in vitro* from the blood precursor cells isolated from critically ill patients, which accumulated p62, and displayed reduced expression of Atg5, Atg7, and LC3–II compared to healthy controls, suggesting deficient autophagy, whilst addition of rapamycin reduced osteoclast formation. PCR arrays revealed a down-regulation of histone methyltransferases coupled with an up-regulation of negative regulators of autophagy. Critically ill rabbits displayed a reduction in trabecular and cortical bone, which was rescued with rapamycin.

**Conclusions:**

Deficient autophagy in osteoclasts and their blood precursor cells at least partially explained aberrant osteoclast formation during critical illness and was linked to global histone hypomethylation. Treatment with the autophagy activator Rapamycin reduced patient osteoclast formation *in vitro* and reduced the amount of bone loss in critically ill rabbits *in vivo*. These findings may help to develop novel therapeutic targets to prevent critical illness-induced bone loss.

## Background

We have previously reported that circulating biomarkers of bone resorption were substantially elevated during critical illness whereas markers of bone formation were low [1]. Such an imbalance may predispose critically ill patients to impaired fracture healing, osteoporosis, and increased risk of new fractures during intensive care unit (ICU) stay or rehabilitation. Indeed, it has recently been shown that female survivors of critical illness display an increased risk of fragility fractures up to 8 years after their stay in the ICU [2]. Interestingly, in an in-house rabbit model of prolonged critical illness trabecular bone mineral density (BMD) and bone mineral content (BMC) were significantly reduced [3]. In addition, analysing osteoclast formation *in vitro* grown from peripheral blood mononuclear cells (PBMCs) isolated from critically ill patients revealed an increase in circulating early osteoclast precursors and the formation of much more mature, resorptive osteoclasts from critically ill patient PBMCs [4]. However, the mechanisms behind this increase in osteoclast formation remain unclear.

The importance of autophagic signaling for osteoclastogenesis was first realized when it was discovered that mutations in the ubiquitin binding protein p62/SQSTM1 was implicated in Paget’s Disease of Bone (PDB), a disease associated with rapid bone remodeling and weakness due to aberrant osteoclastogenesis [5]. More recently, it has been shown that autophagy regulates the lysosomal release of lysosomal components by osteoclasts [6], and pharmacological inducers of autophagy reduce pathological osteoclast formation [7]. We have also recently shown that autophagy is deficient in vital organs and tissues from critically ill rabbits and humans, with an accumulation of p62 and a decrease in the LC3II/I ratio contributing to mitochondrial dysfunction, organ failure, and adverse outcome [8–10]. It is therefore possible that deficient autophagy may also contribute to the increased osteoclast formation and activity during critical illness.

It has recently become apparent that alterations in gene expression alone are insufficient to explain the dynamic and complex process of pathogenic osteoclast formation during metabolic bone disease, and epigenetic regulation is likely to play a role. In particular, the expression of key developmental genes are tightly regulated by methylation of histone H3 lysine 4 (H3K4me3) and lysine 27 (H3K27me3). Interestingly demethylation of H3K27me3 in osteoclast precursors has been associated with the expression of NFATc1, a marker of mature osteoclast formation [11].

Epigenetic factors have also been implicated in regulating autophagy in various pathological conditions [12]. Both epigenetic and autophagic regulation of signalling pathways are known to be highly regulated by environmental factors, particularly during skeletal remodelling [13]. Autophagy activators have shown promising effects against age-related disorders in experimental models [14, 15]. In the clinical setting, the therapeutic value of epigenetic modulators has recently been discovered for conditions such as pre-malignancy and neurodegenerative disorders [16, 15].

We here hypothesize that deficient autophagy, in part related to global histone hypomethylation, may be involved in the increased osteoclast formation and activity during critical illness. We also hypothesize that autophagy inducers could reverse this osteoclast phenotype *in vitro* as well as the resulting bone loss *in vivo* in a rabbit model of critical illness.

## Methods

### *1. In vitro* model of bone resorption during critical illness

#### Experimental subjects

Human peripheral blood was collected from prolonged critically ill patients (n = 6, 24–82 years of age, mean age 51 ± 20.1 years of age) and healthy control volunteers, matched for age, sex, and body mass index (BMI) (n = 6, 25–90 years of age, mean age 52.9 ± 23.1 years of age) (Table 1). Prior to sample collection, patients who were in the ICU for 7 days or longer were selected by excluding those who had received steroidal drugs or bisphosphonates in the past 12 months.

**Table 1:** Characteristics of critically ill patients and age-, sex, and BMI-matched healthy volunteer controls whose peripheral blood was used to isolate PBMCs for in vitro osteoclast differentiation studies

Characteristic	Patients	Controls	*p*
*n*	6	6	
Age (years), median (IQR)	51 (24–82)	53 (25–90)	0.88
Male gender, %	83 %	83 %	>0.99
BMI (kg/m2), mean ± SD	25.6 ± 5.4	25.4 ± 2.9	0.93
ICU stay upon day of sampling (days), median (IQR)	15.3 (7–39)	n/a	
APACHE-II score upon day of sampling, median (IQR)	27.8 (12–41)	n/a	
WBC upon day of sampling, median (IQR)	14.2 (9–18)	n/a	
CRP upon day of sampling, median (IQR)	143.8 (31–303)	n/a	

All protocols were approved by the Institutional Review Board of the Leuven University (ML6195). Written informed consent was obtained from all healthy volunteers and from the patients or, when the patient was unable to give consent, from the closest family member.

#### Peripheral blood mononuclear cell isolation and culture

Peripheral blood mononuclear cells were isolated using the Ficoll Hypaque density centrifugation technique. For osteoclast differentiation, cells were seeded at a density of 5×10^5^ cells per well [4, 17]. All cultures were performed in quadruplicate in 16–well culture slides (VWR, Leuven, Belgium), and cells were cultured in α–MEM supplemented with 10 % foetal bovine serum (FBS), 100 IU/ml penicillin and 100 μg/ml streptomycin. All cultures were maintained at 37 °C in a humidified atmosphere with 5 % CO2. For the induction of autophagy, cells were cultured in the presence of 1 nM rapamycin (Cayman Chemical, Ann Arbor, MI, USA). After 14 days, PBMCs were fixed, and stained for the osteoclast-specific marker tartrate-resistant acid phosphate (TRAP), and the formation of TRAP positive multi-nucleated (more than three nuclei) cells was quantified by counting the multi-nuclear stained cells in each well [18].

#### Gene expression and protein analysis of osteoclast and autophagy markers

For gene expression analysis, PBMCs were seeded at 100,000 cells/cm^2^ and cultured for 14 days in α–MEM supplemented with 10 % FBS, 100 IU/ml penicillin and 100 μg/ml streptomycin. Total RNA was isolated using the RNeasy kit (Qiagen Benelux, Venlo, Netherlands), and cDNA was synthesized with the SuperScript III First Strand synthesis system for real-time PCR (Invitrogen). Quantitative Real-Time SYBR Green (Invitrogen) PCR was performed for osteoclast markers TRAF6, CD16 and NFATc1, and autophagy markers SQSTM1, Atg3, and Atg7 according to the manufacturer’s protocol, with mRNA levels normalized to β-actin expression. SYBR Green qPCR primers were designed to span an intron so that only RNA-specific amplification was possible (See Table 2 for primer sequences). Total RNA samples subjected to cDNA synthesis reactions in the absence of reverse transcriptase were included as negative controls and relative differences in expression were calculated using the 2^-∆CT^ method [19]. For protein expression analysis, PBMCs were seeded at 100,000 cells/cm^2^ and cultured for 14 days in α–MEM supplemented with 10 % FBS, 100 IU/ml penicillin and 100 μg/ml streptomycin. After 14 days the cell monolayer was washed with ice-cold 1xPBS, and lysates were prepared by adding lysis buffer containing protease and phosphatase inhibitors. Lysates were scraped into a 1.5 ml microcentrifuge tube, and centrifuged for 15 min at 4 °C. Supernatants were transferred to fresh microcentrifuge tubes and stored at −80 °C. The protein concentration was determined by the Pierce BCA Protein Assay (VWR, Leuven, Belgium). Equal amounts of protein were loaded onto each lane of a 4–12 % Bis-Tris gel and subjected to electrophoresis under reducing conditions. After blotting, polyvinylidene difluoride membranes were blocked for one hour (5 % milk powder in 0.1 % PBS/Tween) and incubated with the appropriate antibody overnight at 4 °C. Antibodies were purchased from Abcam, Cambridge, UK (p62, LC3, Beta-Actin), or Cell Signaling Technology, Danvers, MA (Atg5, H3K9, H3K27, H3). Protein levels were normalized to the median control levels. Binding of the secondary antibody was visualized by enhanced chemiluminescence (ECL).

**Table 2:** Primer sequences for qPCR analysis

Gene name	Forward (5’-3’)	Reverse (3’-5’)
**Human**		
TRAF6	GCAAGTATGAATGCCCCATC	TGGACATTTGTGACCTGCAT
CD16B	CATTCCAAAAGCCACACTCA	CCCAGGTGGAGAGAATGATG
NFATc1	GCATCACAGGGAAGACCGTGTC	GAAGTTCAATGTCGGAGTTTCTGAG
SQSTM1	CGTCTGCCCAGACTACGACT	GTGTCCGTGTTTCACCTTCC
ATG3	CCACGATTATGGTTGTTTGG	GTGGCAGATGAGGGTGATTT
ATG7	ACCTTGGGTTGCAATGTAGC	CTTACCACCCCCTAGGCAAT
**Rabbit**		
RUNX2	CTTCACAAATCCTCCCCAAG	ATGCGCCCTAAATCACTGAG
OSX	ATGGCGTCCTCTCTGCTTG	GCTTCTCGTGCCTGCTTT
COLA1	Life Technologies (Paisley, UK): Reference number. OC 03 396116_A 1	
OPG	GTGCACTTGGTTTGCTGCTA	CCTGAAGAATGCCTCCTCAC
RANKL	AAAATGAAGATTTGCAAGACG	TTTCTCGGCTCTCAGGTGTT
DAP12	CTGGTGTTGACCCTGCTCAT	TGACTCGGTCTCAGTGATGC
CD16B	GACCTCCACATTCCAGAAGC	GAGAACGGAGGACGAGATGA

#### Analysis of global histone methylation status and interaction with autophagy signaling in patient osteoclasts

Global methylation of histone H3 lysine 9 (H3K9me3) and lysine 27 (H3K27me3), which both result in global repression of gene expression, was compared to total histone and beta-actin in freshly isolated PBMCs and mature osteoclasts from healthy controls and critically ill patients by Western blotting. In an attempt to elucidate the role of autophagy and histone modifications in aberrant osteoclast formation in critically ill patients, the SABioscience RT2 Profiler™ PCR Arrays (Qiagen Benelux) for Autophagy and Epigenetic Chromatin Modification Enzymes were utilized. These qPCR arrays profile the expression of 84 key genes representative of autophagy and epigenetic chromatin modifications. PBMCs were cultured for 14 days with 10 % FBS, and total RNA was isolated using the RNeasy kit (Qiagen Benelux) as previously described. The single strand cDNA from 1 μg total RNA was synthesised using RT2 first strand kit (SABioscience). Real-Time PCR was performed according to the User Manual of RT2 Profiler PCR array system (SABioscience) using SYBR Green PCR Master Mix in a StepOnePlus PCR system (Applied Biosystems). Data analysis was carried out using the online SABioscience Array data analysis software, and genes were identified as up- or down-regulated with a 2–fold or higher change in expression. Using Ingenuity Pathway Analysis (IPA) software, networks for >2–fold down-regulated epigenetic genes and up-regulated autophagy genes were created.

### *2. In vivo* rabbit model of bone loss during critical illness

#### Animals

The study protocol was approved by the KU Leuven Ethical Review Board for Animal Research (P108/2009). All animals were treated according to the Principles of Laboratory Animal Care (U.S. National Society for Medical Research) and the Guide for the Care and Use of Laboratory Animals (National Institutes of Health). In our animal model, critical illness was induced by burn injury, which has previously shown to reveal the dynamic endocrine and metabolic changes characteristic of critical illness, including hyperglycemia, and endogenous insulin deficiency. This model has previously been characterized and validated as a robust *in vivo* model of critical illness [20]. At day 0, adult, 3– to 4–month-old male New Zealand White rabbits were anesthetized and catheters were inserted in the right jugular vein and right carotid artery, allowing intravenous infusion of fluids and repetitive blood sampling, respectively. Thereafter, a paravertebral block was performed using lidocaine (Xylocaine, AstraZeneca, Brussels, Belgium), and a full thickness third-degree burn injury was inflicted on the flanks (*n* = 12) (15–20 % body surface area; painless by itself as cutaneous nerves for sensation of pain are destroyed). Immediately after burn injury, rapamycin-treated animals (n = 6) received an intramuscular injection of 0.06 mg/kg rapamycin (Rapamune; Pfizer Limited, Sandwich, UK) followed by a 0.05–mg/kg injection after 24 and 48 hrs. During the first day, animals received fluid resuscitation consisting of Hartmann enriched with 5 % glucose (Baxter, Lessines, Belgium), which was replaced by parenteral nutrition (33.3 % Oliclinomel N7 (Baxter), 33.3 % Hartmann, and 33.3 % Glucose 50 %) at day 1. Animals were targeted to hyperglycemia throughout the 3–day study period, which was selected to ensure autophagy-related changes were observed. This group was compared with a similarly treated group not receiving rapamycin (n = 6) and with healthy animals (n = 4). All animals survived until day 3 and were anesthetised and sacrificed by cardiectomy. The left and right tibiae were dissected, dipped in polyvinyl alcohol, and snap-frozen in liquid nitrogen, and stored at −80 °C until further analysis.

#### Bone quantification and gene expression

Peripheral quantitative computed tomography (pQCT; Stratec XCT Research densitometer, Norland Medical Systems, Fort Atkinson, WI, USA) was used to assess the cortical area and strength-strain index. Using a voxel size of 0.070 mm, slices of 0.2 mm thickness were obtained. Three metaphyseal scans were taken 2.4 mm from the proximal end of the tibia and an inner threshold was set to 30 % of the total cross-sectional area to define the bone region. Following pQCT, the proximal tibiae of these rabbits were decalcified in 10 % ethylenediaminetetraacetic acid (EDTA) at 4 °C in order to enable sectioning of tissues [16]. After 6 weeks, the tibiae were processed to paraffin and sections of 5 μm thickness were cut and stained for H&E, and the amount of trabecular bone quantified by ImageJ® software (National Institutes of Health) [4]. Gene expression analysis was performed on the trabecular bone of the right tibia of critically ill rabbits as previously described [3].

#### Statistical analysis

The data were processed using the Statistical software package StatView 5.0.1 (SAS Institute Inc.). Student’s *t* test was used for the comparison of normally distributed data (presented as mean ± SD), and the Mann–Whitney *U* test for data that were not normally distributed (presented as median and IQR, unless otherwise indicated). P values less than or equal to 0.05 were considered statistically significant. Statistical significance is indicated on all graphs as follows: *: p < 0.05, **: p < 0.01, ***: p < 0.001.

## Results

### Autophagy is deficient in PBMCs and osteoclasts from critically ill patients

**Fig. 1:**
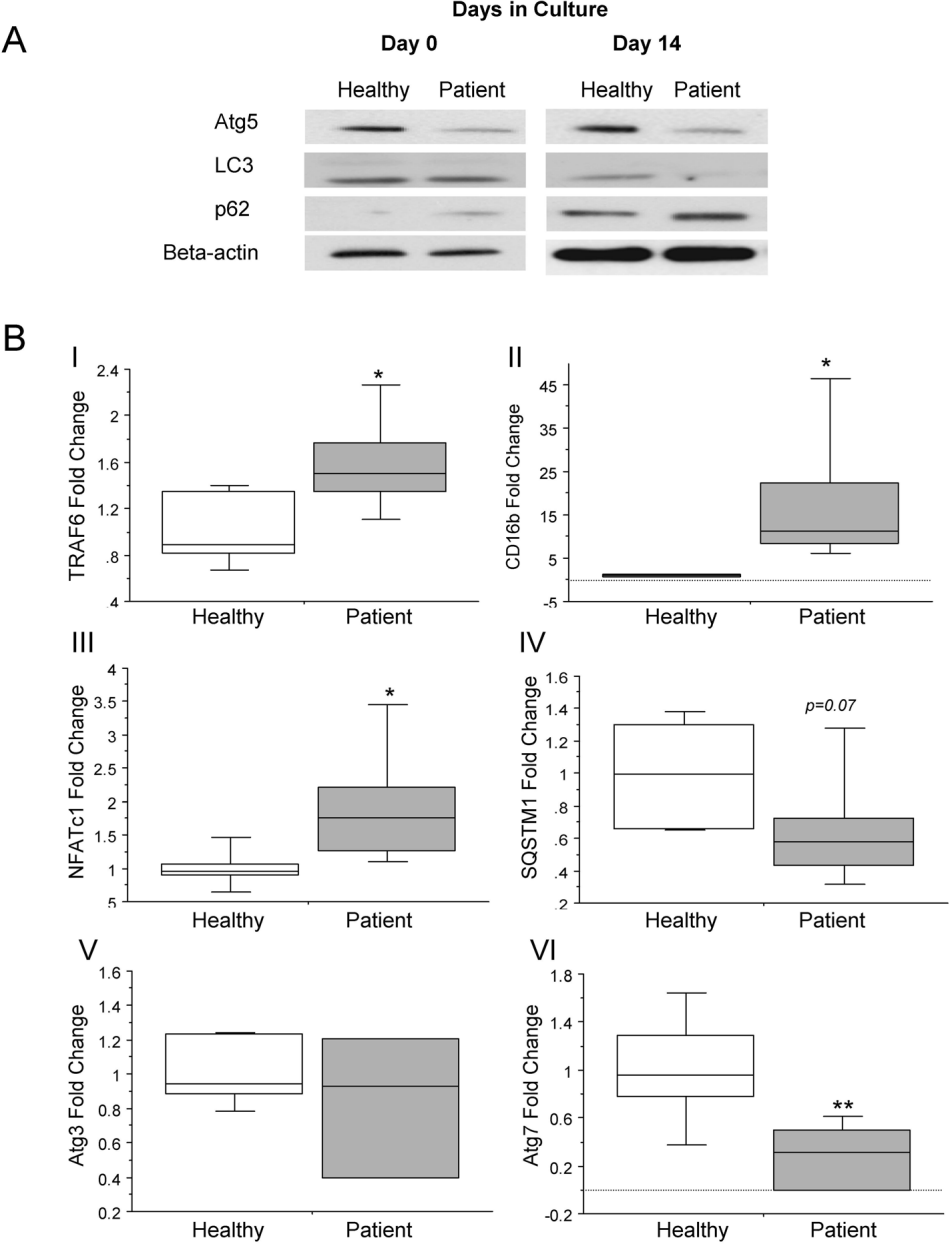
Deficient autophagy in critically ill patient PBMCs. (**a**) Undifferentiated PBMCs from critically ill patients displayed decreased levels of Atg5 and increased levels of p62 protein, suggesting autophagy was already deficient in these cells. In PBMCs grown for 14 days in culture, protein levels of both Atg5 and LC3–II were decreased, in combination with an accumulation in p62. (**b**) At the level of gene expression, markers of mature osteoclast differentiation (TRAF6, CD16B, NFATc1) were significantly increased in osteoclasts from critically ill patients compared to healthy controls (1.6-fold, 17.5–fold, and 1.9–fold, respectively; p < 0.05). Expression of autophagy markers SQSTM1, the gene encoding p62, and Atg3 were not significantly different between cell populations, however, Atg7 was significantly decreased by 3.5–fold in patient cells compared to healthy controls (p < 0.01) (n = 3; *p < 0.05; **p < 0.01)

Freshly isolated PBMCs and PBMCs grown in culture for 14 days were analysed for the expression of markers of autophagy in critically ill patients compared to matched healthy controls. Freshly isolated PBMCs *(Day 0 of culture)* from critically ill patients displayed lower levels of Atg5 protein than those from healthy controls. Levels of LC3–II were unchanged, and an accumulation of p62 protein was observed, suggesting autophagy was already supressed in these non-cultured precursor cells. Additionally, in PBMCs which had been differentiated in culture for 14 days, protein levels of both Atg5 and LC3–II were decreased when compared to cultured cells from healthy controls. In addition, p62 protein levels were increased, suggesting an accumulation in p62 (Fig. 1a). Unsurprisingly, markers of mature osteoclast differentiation (TRAF6, CD16B, NFATc1) were significantly increased in mature cultured osteoclasts from critically ill patients compared to healthy controls (1.6–fold, 17.5–fold, and 1.9–fold, respectively; p < 0.05; Fig. 1b, I, II, and III). Expression of autophagy genes SQSTM1 which is the gene encoding p62, and Atg3 were not significantly different between cell populations, whereas Atg7 was significantly lower, by 3.5–fold, in patient cells compared to healthy controls (p < 0.01; Fig. 1b, IV, V, and VI).

### Addition of the autophagy inducer rapamycin to critically ill patient PBMCs *in vitro* leads to decreased osteoclast formation

**Fig. 2:**
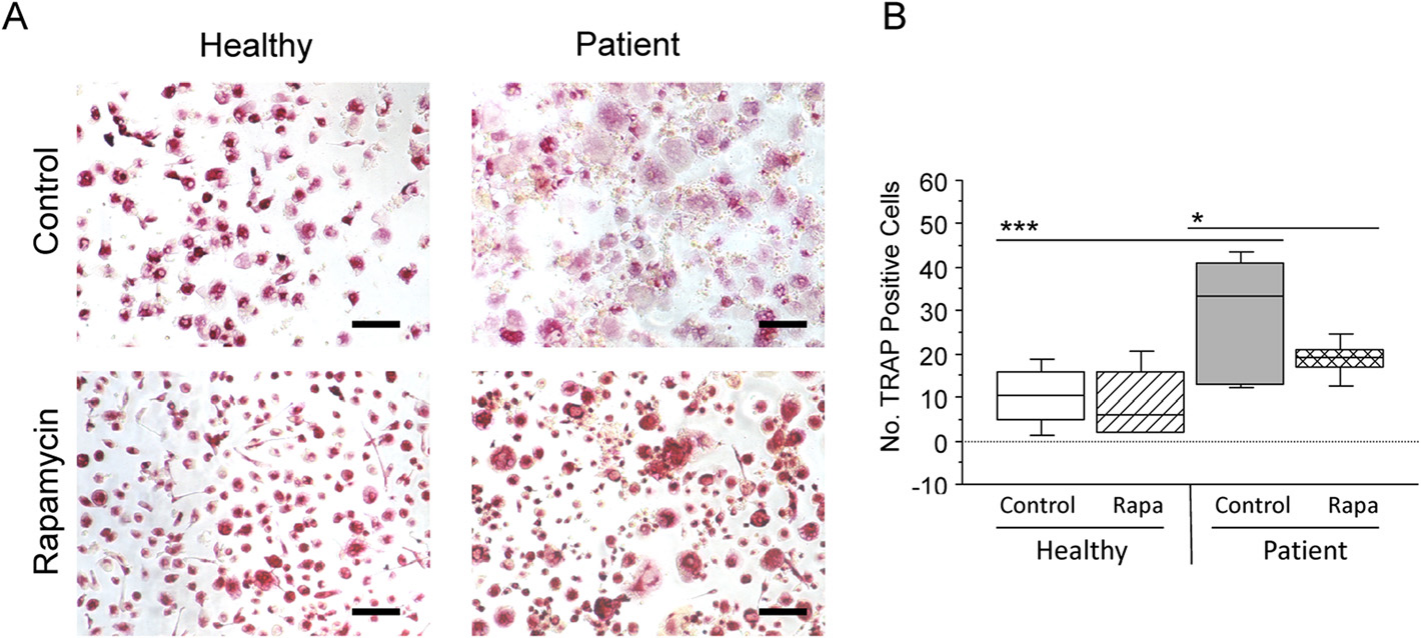
Induction of autophagy *in vitro* leads to decreased osteoclast formation in critically ill patient PBMCs. (**a**) The formation of mature, multi-nuclear (≥3 nuclei, tartrate-resistant acid phosphatase (TRAP) positive) osteoclasts was quantified visually using ImageJ software. (**b**) After 14 days in culture, osteoclast formation increased by 65 % in PBMC cultures from critically ill patients than those from healthy controls. The addition of rapamycin did not alter osteoclast formation in healthy control cultures, however, in patient cultures, osteoclast formation was reduced by 36 % (n = 6; *p < 0.05; ***p < 0.001)

To further examine the role of autophagy in bone loss in an *in vitro* model relevant for the human condition of critical illness, PBMCs were isolated, and pooled from an age-, sex-, and BMI-matched set of critically ill patients and healthy controls, and analysed for osteoclast formation in the presence of the autophagy inducer rapamycin. After 14 days of culture, the formation of mature, multi-nuclear (≥3 nuclei, TRAP positive osteoclasts was increased by 65 % in PBMC cultures from critically ill patients than those from healthy controls (Fig. 2a and b; p < 0.001). The addition of rapamycin reduced osteoclast formation by 36 % in patient cultures (Fig. 2a and b; p < 0.05).

### Aberrant epigenetic modifications are linked to alterations in the autophagy pathway in critically ill patient osteoclasts

**Fig. 3:**
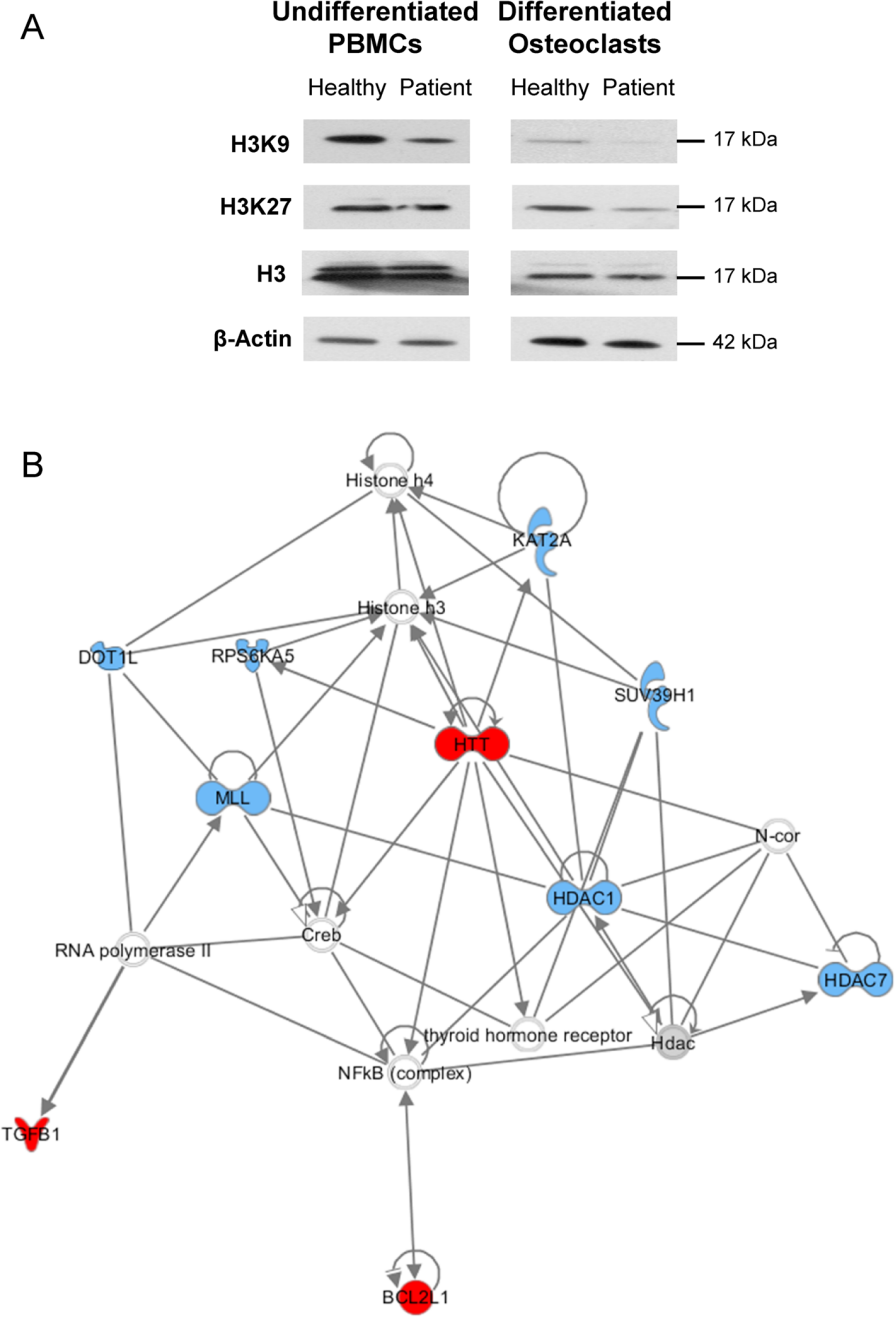
Aberrant epigenetic modifications are linked to alterations in the autophagy pathway in critically ill patient osteoclasts. (**a**) Global histone methylation at histone H3 methylated Lys9 (H3K9) and histone H3 methylated Lys27 (H3K27) was decreased in freshly isolated PBMCs and mature osteoclasts from critically ill patients compared to healthy controls, whilst levels of unmethylated histone H3 were unchanged. (**b**) Using the Ingenuity Pathway Analysis site, a number of down-regulated chromatin modification enzymes (namely DOT1L, RPS6KA5, MLL, KAT2A, SUV39H1, HDAC1, and HDAC7) could be linked to up-regulated genes related to inhibition of autophagy (HTT and BC2L1), suggesting that histone hypomethylation may be associated with deficient autophagy in critically ill patient osteoclasts (red network hubs = autophagy-related genes; blue network hubs = chromatin modification-related genes)

In PBMCs and mature osteoclasts from critically ill patients, global histone methylation at histone H3 methylated Lys9 (H3K9) and histone H3 methylated Lys27 (H3K27) was lower than in those from healthy controls, whilst levels of unmethylated histone H3 were unchanged (Fig. 3a). The autophagy PCR array revealed that from a total of 84 key genes, 10 genes were up-regulated by 2–fold or more and 12 which were down-regulated in patient osteoclasts compared to healthy controls (Table 3). The epigenetic modification array analyzing osteoclast RNA revealed up-regulation of only one gene (PRMT8, 13.4–fold), whilst 21 genes were down-regulated by 2–fold or more (Table 4). Using the Ingenuity Pathway Analysis site, a number of down-regulated chromatin modification enzymes (namely DOT1L, RPS6KA5, MLL, KAT2A, SUV39H1, HDAC1, and HDAC7) could be linked to an up-regulation of genes that are related to inhibition of autophagy (HTT and BC2L1) (Fig. 3b).

**Table 3:** Change in expression of autophagy-related genes in critically ill patient osteoclasts

Gene Name	Fold Change
HTT	4.02
ATG9A	3.79
GAA	3.67
LAMP1	3.08
EIF4G1	2.77
DAPK1	2.64
RGS19	2.43
ATG16L2	2.15
TGFB1	2.08
BCL2L1	2.01
TMEM74	−6.56
IRGM	−5.52
IGF1	−5.44
TNF	−4.73
ATG10	−3.35
IFNG	−3.32
PTEN	−3.31
CXCR4	−3.16
FAS	−2.84
PRKAA1	−2.81
MAP1LC3B	−2.73
GABARAPL2	−2.06

**Table 4:** Change in expression of epigenetically-related genes in critically ill patient osteoclasts

Gene Name	Fold Change
PRMT8	+13.45
PRMT6	−11.22
HDAC7	−5.25
KDM5C	−4.00
HDAC11	−3.86
HDAC10	−3.54
DNMT3A	−3.33
KDM6B	−3.33
MLL	−2.98
DNMT3B	−2.78
USP21	−2.58
RPS6KA5	−2.55
SETD6	−2.53
SUV39H1	−2.42
PRMT1	−2.35
WHSC1	−2.25
DOT1L	−2.22
AURKB	−2.18
DZIP3	−2.17
HDAC1	−2.12
KDM4A	−2.03
KAT2A	−2.00

### Addition of the autophagy inducer rapamycin restores bone loss in critically ill rabbits *in vivo*

**Fig. 4:**
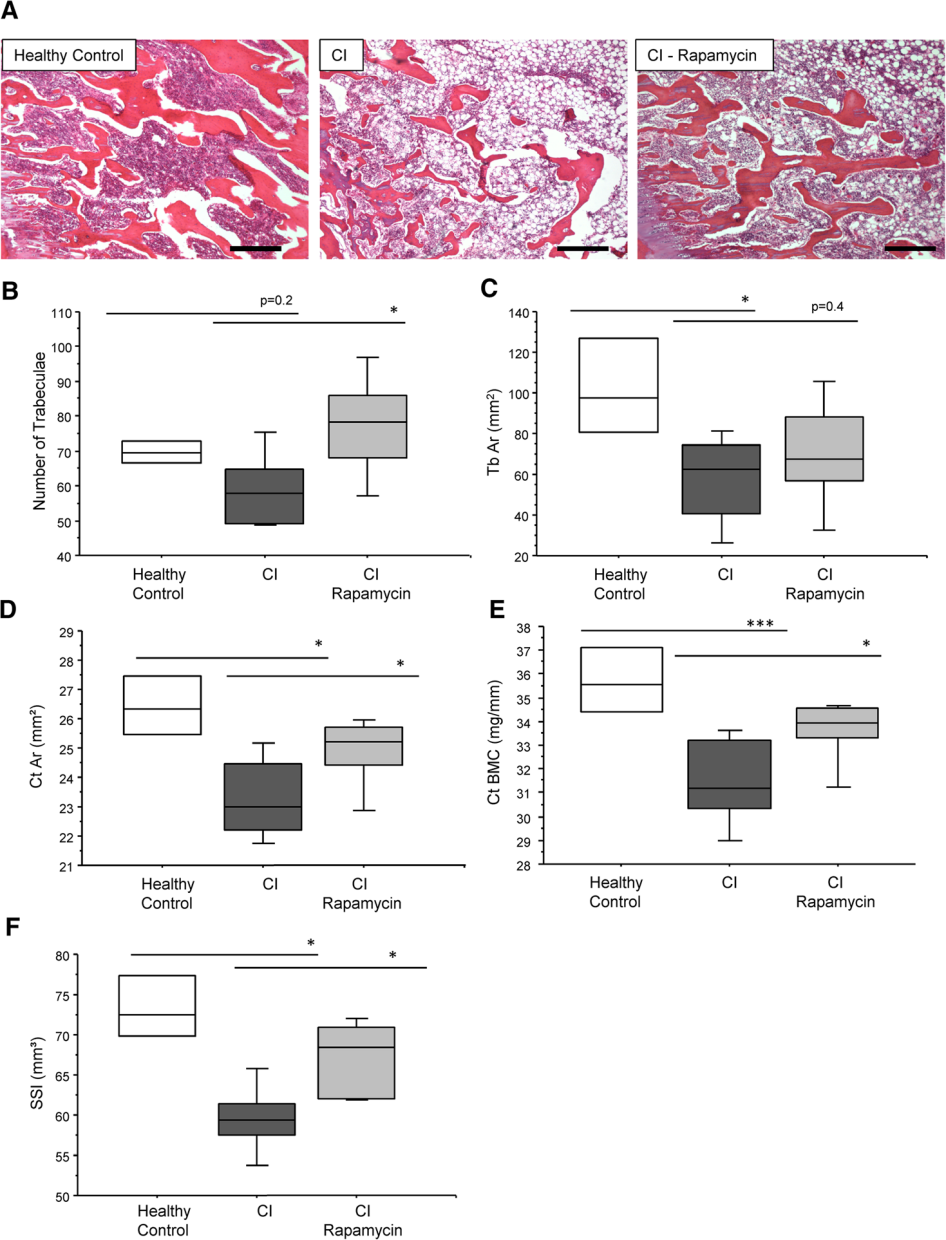
Induction of autophagy restores bone loss in critically ill rabbits. (**a**) After 3 days, there was a trend (p = 0.2) for a reduction in number of trabeculae (**b**) and a significant reduction in trabecular area (**c**) in critically ill rabbits compared to controls. Similarly, the cortical area (**d**) cortical bone mineral content (BMC) (**e**) and strength strain index (SSI) (**f**) was significantly reduced. Critically ill rabbits treated with rapamycin displayed a significantly higher number of trabeculae, cortical area, and SSI compared to untreated sick rabbits (p < 0.05; n = 6 per group)

In order to examine the direct effect of an autophagy inducer in critically ill rabbits, trabecular, and cortical area were analysed in sick rabbits treated with rapamycin or saline. After 3 days, the number of trabeculae was not significantly lower in critically ill rabbits compared to controls (15.3 % (p = 0.20); Fig. 4a). The trabecular area, however, was reduced by 44.1 % (p ≤ 0.05), (Fig. 4b). The cortical area and bone mineral content (BMC) from sick rabbits were significantly lower than in controls (12.1 % (p ≤0.05) and (12.2 % (p ≤ 0.05) respectively (Fig. 4d and e)), as was the Strength-Strain Index (SSI) (16.1 % (p ≤0.05), Fig. 4f. Rapamycin treatment of critically ill rabbits resulted in a significantly higher number of trabeculae (31.1 %), increased cortical area (6.9 %), BMC (6.9 %), and SSI (13 %) compared to untreated critically ill rabbits (Fig. 4a–f).

**Fig. 5:**
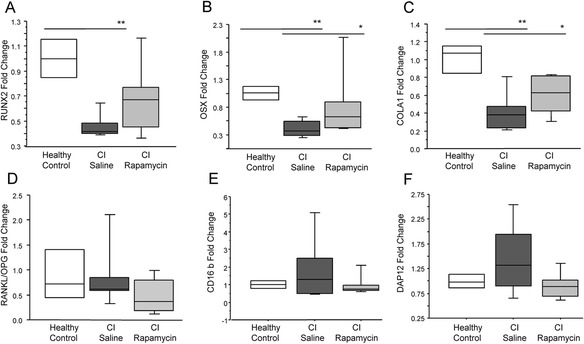
Induction of autophagy restores the expression of osteoblast differentiation markers in trabecular bone of critically ill rabbits. The expression of RUNX2 (**a**), OSX (**b**), and COLA1 (**c**) was significantly decreased in the critically ill rabbits as compared to healthy controls (p < 0.05; n = 6 per group). (**d**) No change in the RANKL/OPG ratio was observed critically ill rabbits, however a trend for increased CD16b (**e**) and DAP12 (**f**) expression was observed (n = 6 per group)

To investigate the effect of rapamycin on osteoblast differentiation the gene expression of RUNX2, OSX, and COL1A1 were measured in the trabecular bone of critically ill rabbits treated with rapamycin or saline and in healthy control rabbits. The expression of these 3 markers was significantly lower in the critically ill rabbits than in healthy controls (RUNX2: 54 % (p ≤ 0.01), OSX: 64.3 % (p ≤ 0.01) and COL1A1: 57.9 % (p ≤ 0.01) (Fig. 5a–c). Administration of rapamycin significantly increased OSX (p ≤ 0.05) and COLA1 (p = 0.04) expression compared to critically ill rabbits treated with saline.

Osteoclast differentiation in trabecular bone was assessed through the gene expression of markers of 2 alternative signalling pathways attributing to osteoclastogenesis, RANKL/RANK/OPG, and CD16, as we have previously shown that CD16 signalling is essential for osteoclastogenesis during critical illness [3]. No change in the RANKL/OPG ratio was observed critically ill rabbits as previously reported (Fig. 5d), however a trend for increased CD16b and DAP12 expression was observed (89.5 % and 45.1 %, respectively), which was reversed with rapamycin (Fig. 5e and f).

## Discussion

In the current study we have investigated the role of autophagy in critical illness-induced bone loss, and the potential role of epigenetic changes. The cellular control of autophagy in human critical illness was studied *in vitro*, where PBMC osteoclast precursors in the blood of critically ill patients displayed markers of supressed autophagy, which was maintained upon differentiation into mature osteoclasts. Furthermore, treatment with the autophagy inducer rapamycin resulted in an inhibition of pathological osteoclast formation from patient PBMCs. Interestingly, global histone methylation was reduced in patient PBMCs and osteoclasts, and this was associated with an increase in the expression of autophagy-inhibiting genes such as BCL2L1. It is known that female survivors of critical illness display increased fracture risk up to 8 years following ICU discharge [2], and we have previously shown that trabecular bone is lost in a rabbit model of critical illness [3, 4]. In the current study, we have expanded these findings by showing that the administration of an autophagy inducer ameliorates critical illness-induced bone loss.

We have previously shown that autophagy is insufficiently activated in liver, kidney, and skeletal muscle of critically ill rabbits [3, 8]. Additionally, in critically ill human patients, autophagy appears insufficiently activated to adequately clear cell damage in skeletal muscle. By tolerating an important macronutrient deficit for one week in the ICU, however, this insufficient activation was ameliorated [21, 22]. Pharmacological activation of autophagy may be a strategy to improve outcome, as also shown in our animal model of critical illness [8]. The importance of adequately functioning autophagy for normal osteoclast differentiation is apparent in Paget’s Disease of Bone, where mutations in the ubiquitin binding protein p62/SQSTM1 results in aberrant osteoclastogenesis [5]. In the current study, in addition to an increase in markers of osteoclast differentiation, we observe a reduction in Atg5 and Atg7 expression, coupled with reduced LC3–II expression. This suggests a suppressed activation of autophagy, which is supported by reduced SQSTM1 gene expression. Interestingly, it has previously been shown that Atg5 and Atg7 are required for the active function of osteoclasts as bone-resorbing cells, but not for osteoclast formation [6]. Therefore, we cannot exclude the possibility that the decrease in Atg enzymes observed in the current study combined with increased osteoclast differentiation are an *in vitro* phenomenon. However, the observation of an accumulation in p62 protein in patient osteoclasts reinforces the hypothesis that the autophagy process is deficient during critical illness.

Induction of autophagy with the mTOR inhibitor rapamycin significantly reduced aberrant osteoclast formation in patient PBMC cultures, but had no effect on healthy control PBMCs, supporting the hypothesis that deficient autophagy plays a key role in the extreme osteoclast formation observed in critical illness.

The epigenetic regulation of autophagy has recently been highlighted as a key factor in cell survival. In particular, methyltransferases such as G9a have been shown to coordinate the transcriptional activation of key regulators of autophagosome formation by remodeling the chromatin landscape [23]. In the current study, global hypomethylation was observed at H3K9 and H3K27 in patient PBMCs and osteoclasts *in vitro*. The expression of key developmental genes is often regulated by H3K9 and H3K27 hyper- or hypomethylation, and in mature osteoclasts, H3K27 at the promoter region of NFATc1 gene is markedly hypomethylated [11]. In order to investigate whether altered global histone methylation could be linked with autophagy signaling in critically ill patient osteoclasts, PCR arrays for autophagy, and chromatin modification enzymes were carried out. Interestingly, the most up-regulated gene in the autophagy array was HTT, encoding for the huntingtin protein. Previous studies have shown that deficient autophagy results in a marked increase in HTT protein expression, due to inefficient degradation [24], and blocking autophagy in models of Huntingdon’s disease *in vitro* (in adrenal medulla cells or striatal cells) raised levels of exogenously expressed HTT, reduced cell viability, and increased the number of cells bearing mutant HTT aggregates [14]. Importantly, when down-regulated genes from the chromatin modification PCR array were combined with up-regulated genes from the autophagy array, a network was created with HTT appearing as a key network hub gene, in addition to BCL2L1, which inhibits autophagy by binding to the BH3 domain of beclin 1 [25]. These genes were associated with a number of down-regulated methyltransferases such as SUV39H1, MLL, and DOT1L, in addition to histone deacetylases HDAC1, HDAC7, and KAT2A. However, whether hypomethylation is the direct cause of the up-regulation of both HTT and BCL2L1 cannot be elucidated from this data, and additional studies are required to confirm whether there is a direct link between deficient autophagy and epigenetic alterations in aberrant osteoclast formation during critical illness.

We have previously shown that rabbits made critically ill for one week displayed bone loss and displayed signs of insufficiently activated autophagy in other organs and tissues [3, 21]. In the current study, administration of the mTOR inhibitor rapamycin was protective against signs of trabecular and cortical bone loss after only 3 days. In addition, reductions in the expression of osteoblast differentiation markers were also partially restored with rapamycin, whilst trends towards an increase in immune-related osteoclastogenic markers CD16b and DAP12 in sick rabbits were reduced with rapamycin. Interestingly, it has recently been shown that autophagy is induced in osteoblasts during mineralization, and that autophagy deficiency reduces mineralization capacity [26]. Furthermore, a recent study has reported a similar finding in mice with giant cell tumor of bone (GCT), a benign type of tumor with hyperactive multinucleated giant osteoclasts which can cause local osteolytic lesions therefore increasing morbidity. The treatment of GCT mice with rapamycin resulted in reduced osteoclast formation by switching the expression of C/EBPβ isoforms [7]. Similarly, arthritic mice treated with rapamycin or everolimus, an alternative mTOR inhibitor, displayed an improvement in arthritic symptoms and reduced osteoclast formation suggesting rapamycin could be a promising therapy for metabolic bone disease [27]. However, one major caveat with the use of rapamycin is it's immunosuppressant properties, which make it unsuitable for the treatment of critically ill patients. In addition, we cannot exclude that the reduced osteoclast formation *in vitro* and improved bone phenotype observed in critically ill rabbits treated with rapamycin is due to the immunosuppressive effects of rapamycin rather than its autophagy-inducing properties. In addition, we cannot exclude the possibility that the bone-sparing effects observed with rapamycin are not due to direct effects on osteoclasts and osteoblasts in the trabecular bone, but are secondary to alterations in the immune system, inflammation, or angiogenesis. Consequently, further work is required to identify therapies that induce autophagy but do not cause immunosuppressant side effects in critically ill patients.

## Conclusion

The present study has confirmed that PBMCs from critically ill patients are predisposed to form an increased number of mature osteoclasts compared to PBMCs from healthy controls. This increase in osteoclast formation is associated with deficient autophagy, which may be mediated by global histone modifications. Treatment with the autophagy inducer rapamycin reversed these findings *in vitro* and in an *in vivo* rabbit model of critical illness, reduced the amount of bone loss. These findings may help to develop novel therapeutic targets to prevent critical illness-induced bone loss.
